# A Co-Wife for the Cow: Gender Dimensions of Land Change and Livelihood Shift among Loita Maasai of Southern Kenya

**DOI:** 10.1007/s10745-018-0034-7

**Published:** 2018-11-17

**Authors:** Miriam O. Westervelt

**Affiliations:** University College London, London, UK

**Keywords:** Maasai, Gender, Loita Forest, Kenya, Land change, Livelihood diversification

## Abstract

Gender dimensions are fundamental to human/environment systems. I use gender to investigate land change in a forested area of conservation concern in the pastoral rangelands of Kenya Maasailand. Mixed methods reveal a narrative arc from the mid-1970s culminating in a transformation of land, livelihood, and culture by 2014. Empirical findings expand current understandings of livelihood shift in Maasailand to include gender dimensions critical to livelihood success. Remotely sensed satellite data and qualitative evidence expose simplistic narratives about environmental conditions in Loita Forest and Maasai women’s social status. I argue that gender deserves more attention in land-change studies because of its linkages to resource utilization and drivers of forest decline around the world.

## Introduction

Remote sensing technology is helping to integrate social, biological, and earth sciences towards improved understanding of human/environment systems. But gender relations and gendered knowledge are undervalued as human dimensions in these efforts, leaving understandings of environmental change incomplete. Gender-sensitive climate change studies are few despite gender-differentiated impacts (USAID 2015). In gender-blind GIS projects, communities and women are often treated as homogenous groups, and gender-differentiated relationships with land are overlooked. According to Rocheleau (1995):

When the gaze begins from space, and when the gaze-from-space is uninformed by the logic of gendered livelihoods and landscapes, then the erasure of women’s place in the mapped spaces is all but certain*.* (p. 463)

I use a gender perspective to examine changes in livelihood and land cover in Loita, Kenya, an area that offers unique opportunities to explore transitions from pastoralism to agro-pastoralism, resource abundance to scarcity, and in women’s social status because traditional relationships with land under communal tenure persist. Changes in legal land tenure are imminent, however, making these gender-sensitive understandings timely for building pathways to social and environmental sustainability (Leach 2016).

### Linking Land Change and Social Dimensions

In pastoral rangelands, remote sensing, census data, household surveys, and interviews are being used to identify land change drivers. In the Mara-Serengeti ecosystem, geospatial mapping of biodiversity, human settlement, and agriculture shows habitat and wildlife declines since the 1970s. These changes appear to be driven by economics, politics, and land tenure (Homewood *et al*. 2001).

Like land change in rangelands, drivers of worldwide forest loss operate as synergies between local activities and underlying causes (Geist and Lambin 2002) including cultural attitudes and behaviors. Understanding these dynamics is critical since global tropical forest cover declined as much as 6% between 1980 and 2010 (Keenan *et al*. 2015). Kenya lost about 25% of its forest cover between 1990 and 2000 with a spiraling disparity between supply and projected needs for timber (GoK 2016).

Quantifying forest loss is complicated by a lack of consistent definition of forest and interpretation of geospatial images of forest cover. Measurements of global forest cover can vary by 6–13% (NASA 2015; Sexton *et al*. 2016), and estimates of global deforestation patterns range from −25% to +64% forest change from the 1990s to the 2000s (FAO 2010; Kim *et al*. 2015). Other factors contribute to discrepancies including shrub lands, mountain ridges, and exotic plantation trees (Tropek *et al*. 2014). For example, with a definition of “forest” as comprising all trees (including bush and plantation trees) of at least 2.5 m in height, Kenya reports annual forest *gain* of 0.1% since 2000 (GoK 2016). Also complicating forest cover estimates are anthropogenic forests, which increase with population density, as well as time lags in forest-savanna transitions due to climatic change (Fairhead and Leach 1998).

Large forest losses in Kenya evident from remote sensing data are due to insecure land tenure, agriculture, and bush encroachment (Pellikka *et al*. 2009). Forests are critical to livelihood sustainability in pastoral rangelands but there are few quantitative studies of change. Unsubstantiated references to undisturbed environmental conditions in the Loita highlands persist in the literature and have served conservation and development goals, but competing interests in grazing, water, biodiversity, timber, and tourism render it unlikely that Loita land and people have been immune to change.

### Linking Livelihood and Gender in Pastoral Lands

East African pastoralists’ gendered roles are changing as people settle. In the past, Maasai women spent less time than men and children on livestock activities. In Kajiado South, *Ilkisongo* (Table 1) Maasai women now contribute more time to livestock production than men as children go to school and sedentarization and cropping increase (Wangui 2008). Shrinking numbers of adults per household, along with men seeking work away from home, also increase women’s labor. Underlying reasons for livelihood diversification in Maasailand are well documented, but empirical evidence of the gender dimensions remains scant.

**Table 1:** Maa words and their meanings

Maa word	Meaning^a^
*aitore*	To command, to rule (e.g., livestock an owner has disposition rights to)
*aitodol*	To show (e.g., livestock an owner has no disposition rights to)
*ilbuluka*	Male circumcision group (*olporror*) established in about 1989
*ilnyang’usi*	Male circumcision group (*olaji*) established in about 1939
*iloitai*	Section of Maasai living mainly in Loita Division in southern Kenya
*ilkerin*	An administrative area located west of Loita Forest in Loita Division.
*ilkiseeyia*	Male circumcision group (*olporror*) established in about 1968
*ilkisongo*	Section of Maasai living in northern Tanzania and southern Kenya
*ilterito*	Male circumcision group (*olaji*) established in about 1926
*kalenjin*	Ethnic group living mainly in west-central Kenya, northern Tanzania and Uganda
*kikuyu*	Largest ethnic group in Kenya
*manyata*	Anglicized version of *emanyata,* sg; *imanyat,* pl. Settlement where male age group events are held; traditional mud/dung hut (*enkaji emodie*)
*moran*	Anglicized version of *olmurrani,* sg, *ilmurrani*, pl.; warrior
*oledardar*	Not found
*oledat*	Trimeria (*Trimeria sp.)*
*oleparmunyo*	Orange climber (*Toddalia asiatica*)
*olgilai*	Teclea (*Teclea nobilis*)
*olkinyei*	Diamond-leaved euclea (*Euclea divinorum*)
*olkonyil*	Shiny-leaf buckthorn (*Rhamnus prinoides*)
*olmaroroi*	Velvet bushwillow (*Combretum mole*)
*olmisigiyioi*	Rhus bush (*Rhus natalensis*)
*oloiboni*	*Oloiboni,* sg, *iloibonok*, pl; prophet
*oloirien*	African olive (*Olea europaea ssp. africana*)
*olotoroniki*	Not found
*olpiripiri*	East African yellowwood (Podocarpus *falcatus*)
*olsinoni*	Fever tea (*Lippia javanica*)
*oltarakuai*	African pencil cedar (*Juniperus procera)*
*orkitolosua*	Myrica, bayberry (*Myrica kilimandscharica*)
*ormorijioi*	Poison arrow tree (*Acokanthera schimperi*)
*purko*	Section of Maasai living in Narok and Kajiado Counties in Kenya
*sonjo*	Bantu-speaking people of northern Tanzania

Botanical and English names of plants are based on literature and not on botanical vouchers. Helpful sources included Dharani (2002), Maundu *et al*. (2001), and Mol (1996). See Westervelt (2017) for complete list of local plants used by men and women

^a^An age-set (*olaji*) includes two circumcision groups, a right-hand group and a left-hand group (*olporror*). Circumcision of a right-hand group may occur 10 years before a left-hand group. Sources of information include Galaty (1992), Kronenberg Garcia (2015), Mol (1996), Spencer (1988), Oltepesi Institute for Maasai Language and Culture, and key informants

The human dimensions of pastoral land use are controversial in part because of incomplete understandings of dry-land ecology that overlook the complex web of highly variable social and biophysical factors (Homewood 2008). The failure or adverse impacts of projects aimed at destocking, evictions, fencing, and land privatization and ranching schemes are attributed to disregarding these complexities and perpetuating outdated paradigms of equilibrium thinking that downplay non-anthropogenic drivers in pastoral ecosystems .

Similarly, feminist political ecologists highlight oversimplified treatments of gender that disregard continually changing dynamics (Nightingale 2006). Pastoralist literature has been criticized for ignoring gender power relations compared to research on agriculture-based societies (Hodgson 2000) in the face of the complex linkages of gender to age, marital status, and material production.

Changes in Maasai women’s social status are similarly oversimplified (Brockington 2001). In general, Maasai society is perceived as patriarchal, and women are treated as children needing protection (Hodgson 1999a, b). Some posit that Maasai women’s status is a consequence of capitalism and British colonial administration when the once-complementary roles of men and women were restructured to women’s disadvantage. Hodgson (2000) interprets pre-colonial records (Merker 1910) as Maasai women enjoying economic independence and shared rights to livestock and decision-making power and sees conversion to a cash economy as leaving women “at a loss” (Talle 1988: 270). Others suggest Maasai women have been dominated by fathers, husbands, and sons for centuries (Waller 1993; Spencer 1988); are agents of change in diversifying rural household economies (Goldman and Little 2015; Smith 2015); have risen from poverty to professional careers with education, mentors, and husbands of their own choosing; and are “pioneering pathways to stronger livelihoods” (Livingstone and Ruhindi 2013, p. 239).

Using empirical data from 1976 to 2014, I address the following research questions: How has the land and culture changed in Loita? Are livelihood activities gendered? How have they changed? And how do they relate to land cover?

### The Land and People of Loita

**Fig. 1:**
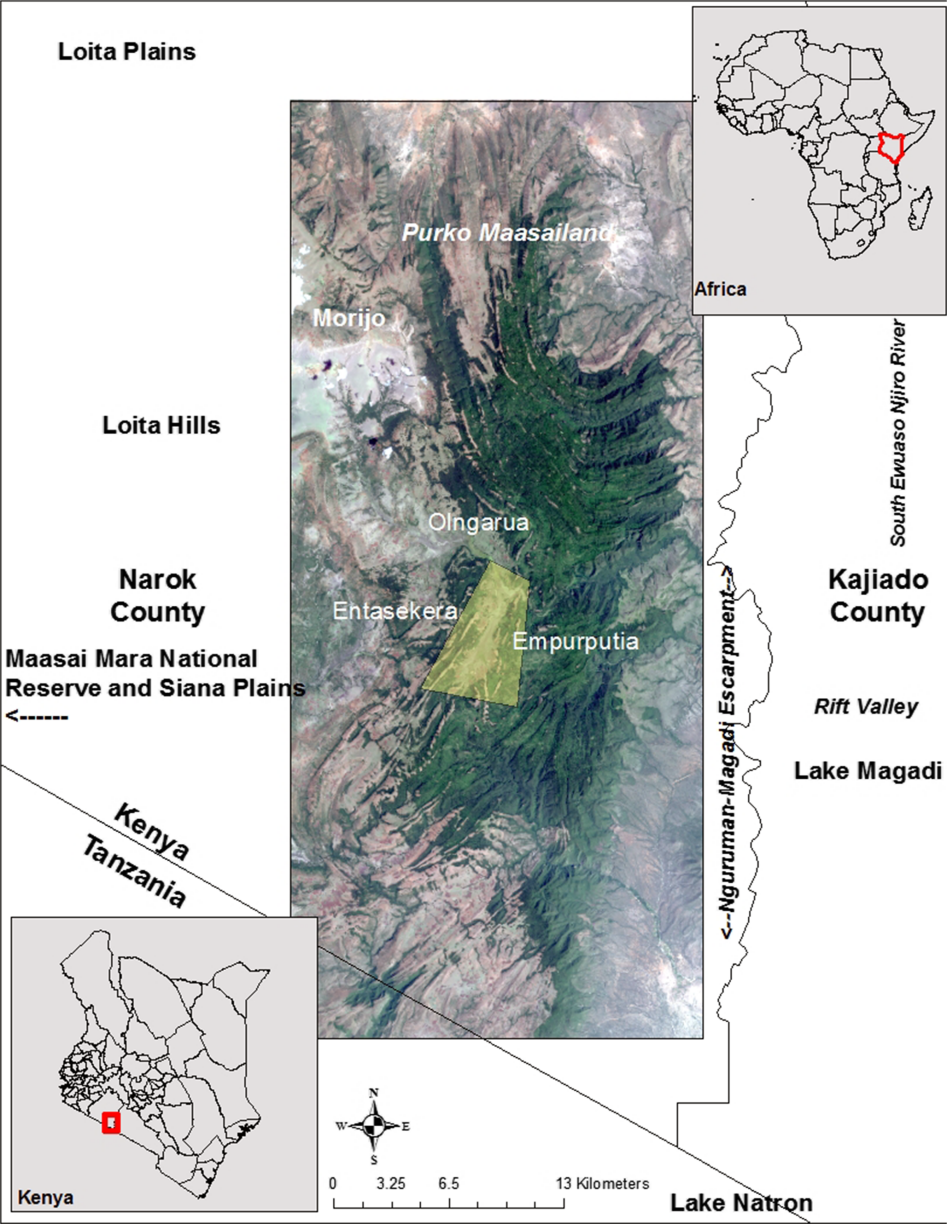
Location of Loita Forest, study area (in yellow), and key features of the surrounding area

Loita Forest is in Loita Division, Narok County, Kenya, about 150 km southwest of Nairobi. Loita includes 1700 km^2^ of diverse ecosystems ranging from arid grasslands to cloud forest and wetlands, with a population of about 25,000 Loita Maasai (*Iloitai*). The core forest extends about 300 km^2^ in the highlands and lacks official boundaries. Kajiado County and the Nguruman-Magadi escarpment lie to the east, Tanzania to the south, Loita and Siana Plains stretch west toward Maasai Mara, and the Loita Hills and *Purko* Maasailand extend north (Fig. 1). The study area in Empurputia extends from the western edge to the center of the forest.

Vegetation consists of grassland, wooded grassland, bushland, thickets on disturbed forest edges, and dry upland forest associations characterized by cedar (*Juniperus spp.),* olive (*Olea ssp.),* and yellowwood (*Podocarpus spp.)* (White 1983; Maundu *et al*. 2001; see also Bussmann 2002). Mean rainfall varies from 600 mm to 2000 mm/year. Rainfall is bimodal, with rains shorter in October–December and longer in March–May.

Understanding of this human/environment system is elusive, with conflicting reports about forest conditions and an absence of empirical data about forest coverage. Betts (1952) described the forest as uninhabited, and Talbot (1960) described a showpiece forest under excellent management by Maasai. Wass (1995) considered it a closed canopy forest, but Maundu *et al.* (2001) reported that as much as 50% of the tree-cover was lost due to local exploitation. Nevertheless Zaal and Adano (2012) still referred to it as one of the last indigenous forests in Kenya that is largely undisturbed. It is now considered an endangered ecosystem and water tower of national importance (KWS 2018) as well as a key area in the Eastern Afromontane biodiversity hotspot (CEPF 2012), hosting globally threatened and restricted-range bird species (BirdLife International 2018).

*Iloitai* are a sub-section of the Maasai people who comprise the largest group of pastoralists in Kenya. Vocabulary, aesthetics in beaded personal ornaments, and strict standards of behavior pertaining to marriage, adultery, wife avoidance at *manyata,* and avoidance between fathers and married daughters (Spencer 1977) distinguish *Iloitai* from other Maasai groups. The forest is vital to their livelihood and culture in providing water, dry season grazing, and habitat for over 250 plants valued for firewood, construction materials, medicinal herbs, veterinary needs, fruits, honey, and ceremonies (Maundu *et al*. 2001). To both Kenyan and Tanzanian Maasai, the Loita Forest is a sacred place where most spiritual leaders (*laibonak)* live. In the past, Maasai moved between traditional wet and dry season areas. Today, customary rules about forest use and grazing rights are breaking down with increased settlement, and impacts on the forest are unclear.

Although *Iloitai* describe themselves as pastoralists, their livelihoods today are highly diversified. Mixed agro-pastoralism expanded in Maasailand in the past century in response to drought, epidemics, commercialization, and land alienation, and gendered livelihood roles are in flux both in the progression to agro-pastoralism (Tanzania: McCabe *et al*. 2010) and in household labor division (Kenya: Wangui 2008). Gender relations and responsibilities are defined by men’s age groups based on circumcision years, and the social position of women, without comparable age groups, depends on marital status and relationships to men (Talle 1988). The male age group system is also the basis of wider social networks customarily governing resource allocation throughout Maasailand (Galaty 1992).

As one of the few indigenous trust land forests remaining in Kenya, Loita is the only territory held communally by Kenya Maasai not yet gazetted or titled by the government into separate private or group parcels. It is also unsurveyed, unlike most of Kenya’s 273 forests. Since the 1970s, the Kenya government, Loita elders, and organizations promoting development and conservation have disputed issues of forest access and ownership. Conflicts fueled by insecure tenure persist, and land claims are “spiraling out of control” (Kronenberg Garcia 2015: 251). Women’s involvement in such disputes is unclear, although wealthy men are known to disperse multiple wives to homesteads across wet and dry-season grazing areas to claim land rights in the event of subdivision.

## Materials and Methods

I used an interdisciplinary mixed methods approach to data collection. I selected households using random stratified proportional sampling and a participatory wealth ranking method (Grandin 1994). I surveyed 30 households (30 husbands and 40 wives) in Empurputia, a rural settlement area of approximately 90 households, with an hour-long structured household questionnaire about changes in livelihood, resource use, and land cover. Shorter and less-structured interviews focused on resource decision making, participatory sketch mapping, and I conducted forest transect walks with an additional 39 men and 41 women. Older women in the sample provided 26 h of oral life histories. Because empirical data on gendered asset allocation among pastoralists are scarce, I adapted Njuki and Mburu’s (2013) measurement of asset disparity to quantify gender differences. Data were collected over a 10-month period with gender congruent field assistants fluent in Maa and English.

A local event calendar helped place informants’ recollections of their land and culture in a historical timeline to minimize pitfalls of retrospective data collection. Establishing approximate birth years was helpful in aligning qualitative data with land changes shown in the satellite imagery. I estimated men’s ages using the male age group system based on circumcision years, supplemented by years of recorded local events (e.g., droughts, floods, fertility blessings). The process was more complex with women. Sometimes younger women knew their age when a particular event happened, but older women generally did not. Helpful questions determining an older woman’s age included “What class were you (or should you have been) in school when you gave birth to this son?” or “Which warriors (*moran)* did you sing for?” since, customarily, girls who were close to circumcision age sang for male peers and slightly older moran.

In constructing the survey questionnaire, attention to local Maa language was important (e.g., the term “cellphone” is *esimu*, but the word *mobile* is used elsewhere; both *aitore* and *aitodol* are verbs referring to terms of cow ownership based on disposition rights (Llewelyn-Davies 1981). To ensure accuracy and consistency, text was translated, pilot-tested, translated back, tested again, and then finalized with educated local Maasai and scholars at the Oltepesi Institute for Maasai Language and Culture. Interviews were audio-recorded and translated into written English by male and female field assistants who cross-checked each other’s work to remove bias. Using Nvivo 10 for Windows software, transcribed text was methodically sorted, coded, and organized into first- (e.g., drivers of change) and second-order (e.g., development) nodes to search for salient information and linked themes with electronic queries and visualization tools (Bazeley and Jackson 2013). Descriptive and inferential statistics for frequency counts and categorical data used Microsoft Excel and the R statistical package. Nonparametric tests (Fisher’s Exact Probability Test for associations between categorical variables, Wilcoxon Mann Whitney for comparing medians, and McNemar test for paired nominal data) were performed since the sample size was small and normal distributions could not be assumed.

Qualitative data provided information about life when and where land change was detected in remotely sensed data. I used Landsat image scenes for 1976 (Landsat 2), 1995 (Landsat 5), and 2014 (Landsat 8) to identify land cover changes (USGS 2016) working with ArcMap version 10.1 (ESRI 2012). Relatively cloud-free (< 10% cloud cover) dry-season months (January–February) were selected for analysis, and the satellite images with the least cloud cover were downloaded. Images were orthorectified in a Universal Transverse Mercator projection with a WGS84 datum, resampled by cubic convolution, and were in GeoTIFF output format. Images were clipped to the extent of Loita Forest (1.571^0^ S, 35.804^0^ W; 1.571^0^ S, 36.011^0^ W; 2.048^0^ S, 36.012^0^ W; 2.048^0^ S, 35.804^0^ W) and Empurputia (1.80529^0^ S, 35.90485^0^ W; 1.81576^0^ S, 35.92515^0^ W; 1.8706^0^ S, 35.86946^0^ W; 1.88001^0^ S, 35.91893^0^ W). The spectral band combination was changed to 4–3-2, which is common in differentiating vegetation types.

Land change data sets were created after rigorous field verification of land classifications. I visited over 40 GPS points in Empurputia and the wider Loita Forest to generate a high-quality training sample. I also consulted official rainfall records (KMD 2014). The classification scheme I used distinguished dense forest (≥ 40% canopy with closed stands of canopy trees ≥5 m tall), light forest/bushland (< 40% canopy with open stands of bushes), grassland (grasses interspersed with woody plants), hydrophytic vegetation areas (mostly wetland plants), and bare land. Polygons representing these classifications were created in ArcGIS. Using post-classification comparison (Singh 1989) to detect change, I compared land coverage for each classification between years. Only relative percentages of land cover were compared with 1976 because of Landsat 2’s coarser instrumentation and lower (60 m versus 30 m) spatial resolution. Percent change in land cover was calculated for 1995–2014.

## Results

### Land Change

**Fig. 2:**
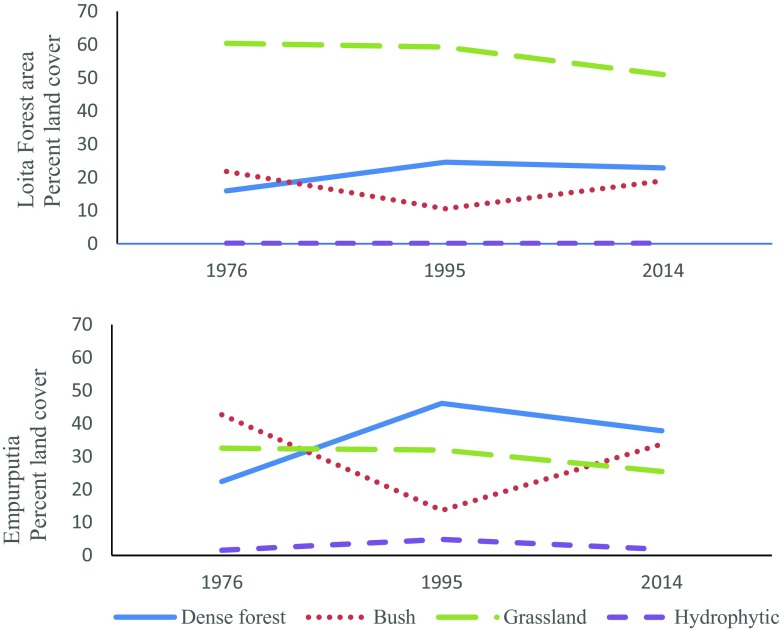
Land cover change in Loita Forest area and Empurputia 1976–2014

**Fig. 3:**
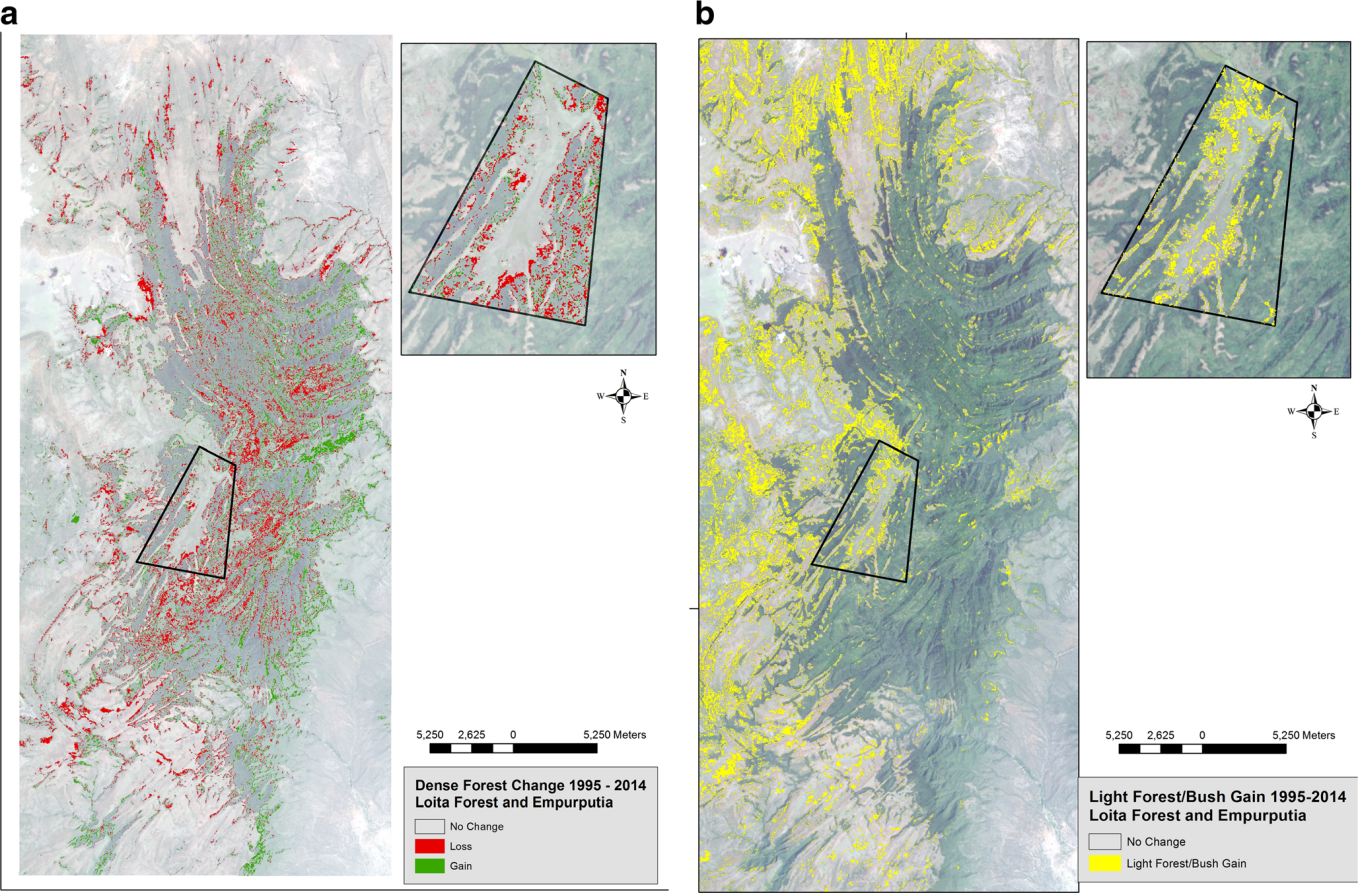
**a**. Dense forest loss (red) in Loita Forest area and Empurputia (black polygon) in 1995–2014 using satellite imagery. **b**. Light forest/bush gain (yellow) in Loita Forest area and Empurputia (black polygon) in 1995–2014 using satellite imagery

Grasslands covered 60% of the Loita landscape in 1976 and declined to 51% in 2014 (Table 2, Fig. 2). There was an obvious negative association between dense forest and light forest/bushland. Bush gained where forest declined and throughout the rangelands below. Between 1976 and 1995, dense forest gained, and bush declined everywhere. In Empurputia, dense forest replaced bush as the dominant classification, covering 46% of the land by 1995. Between 1995 and 2014, dense forest loss was evident everywhere (Fig. 3a), most notably in Empurputia, where it lost 18% cover. In the same period, there was 147% more bush (Fig. 3b) along with 60% loss of wetland vegetation in Empurputia.

**Table 2:** Loita Forest area and Empurputia: Extent of land cover in 1976, 1995 and 2014 and land change between 1995 and 2014

Land cover	1976		1995		2014		Change (1995–2014)	
	km^2^	%	km^2^	%	km^2^	%	km^2^	%
Dense forest								
Loita Forest	197.58	15.97	303.85	24.57	282.44	22.84	−21.41	−7.04
Empurputia	6.84	23.36	14.11	46.10	11.56	37.78	−2.55	−18.07
Light forest/bush								
Loita Forest	269.97	21.82	130.93	10.59	235.09	19.01	104.16	79.55
Empurputia	13.06	42.68	4.19	13.70	10.35	33.81	6.16	147.01
Grassland								
Loita Forest	747.60	60.42	732.98	59.26	630.40	50.97	−102.58	−13.99
Empurputia	9.91	32.37	9.80	32.00	7.79	25.45	−2.01	- 20.51
Hydrophytic								
Loita Forest	2.24	.18	1.82	.15	2.37	.19	.55	30.21
Empurputia	.48	1.56	1.50	4.90	.60	1.96	−.90	−60.00
Bare land								
Loita Forest	19.94	1.61	67.15	5.43	86.40	6.99	19.25	28.66
Empurputia	.32	1.03	1.10	3.30	.31	1.00	−.79	−71.81
Total area								
Loita Forest	1237.33	100.00	1236.73	100.00	1236.78	100.00		
Empurputia	30.61	100.00	30.70	100.00	30.61	100.00

### Gender Dimensions in Periods of Land Change (Table 3)

Years leading up to 1976 were the baseline for exploring change. Talbot (1960: 54–55) described the Loita highlands as a “showpiece” for good management by the Maasai. Loita’s population density was only 4/km^2^ by 1979 (GoK 1981) with Empurputia’s swamp valley and dense forest rarely accessed for fear of dangerous wildlife, mythical ogres, and local legends of Loita Forest known in Maa as “the forest where a little girl was lost” (*entim e naimina enkiyio*) (Mol 1980).

**Table 4:** Open-ended responses to “What is the main way your family pays for what it needs today and the past (10-20 years ago)?” (N = 60)

Main way	Today		Past		McNemar’s test
	Mentions	%	Mentions	%	*P* value
Livestock	38	63.3	55	91.7	.000217
Cultivation	16	26.7	1	1.6	.000001
Off-farm/casual work	6	10.0	4	6.7	.000001
Total	60	100	60	100

**Table 3:** Environment/culture/livelihood linkages over three periods of time based on geospatial data and local informants (*N* = 150)

	Exposition (Before 1976)	Rising Action (1976–1995)	Climax/Transformation (1995–2014)
Environment Land	Showpiece forest; 22% dense forest coverage; productive grasslands	Healthy ecosystem, natural succession; 46% dense forest coverage	18% dense forest loss; 147% bush gain; 21% grassland loss; 60% wetland vegetation loss
Climate		Longer and frequent droughts (1983–1984, 1993–1994, 2000–2001, 2004–2005, 2009)	
Culture Life stage of women in study	Uncircumcised girls (*intoyie*)	Mothers with children (*isiankikin*)	Widows with grandchildren *(intasati*)
Ruling male age groups	*Ilterito* and *Ilnyang’usi*	*Ilnyang’usi* and *Iseuri*	*Iseuri* and *Ilkitoip*
Household decisionmaking	Patriarchal domination; women fear men out of respect (*enkanyit*); women have no power		Men rely on wives as *co-wife for the cow;* interhousehold variability in spousal cooperation/breadwinner effect; gender asset disparity persists
House style/ construction	Men rely entirely on women’s labor to make skin houses and *manyata* houses with pole wood		88% say only men build modern houses today; break forest rules and take live *oltarakua* and *olpiripiri*
Livelihood Strategy	Specialized pastoralism; seasonal migration	92% rely mainly on pastoralism; wives dig small gardens	63% rely mainly on pastoralism; 97% of households cultivate; 27% rely mainly on crop income
Fire	Moderate burning by men		Less burning
Food and fuelwood	Milk, meat, blood, diet; little cooking; women easily access dead wood	Maize meal added to diet; more trips for more fuelwood to cook and clean	Women travel deeper into forest to find preferred *oloirien* fuelwood; break forest rules and take live trees
Main forest activities of females	*Olpul* with moran; gathering fruits and berries	Fuelwood; house building materials; medicinal plants for children	Fuelwood; medicinal plants for children
Main forest activities of males	*Olpul* with uncircumcised girls; herding	Age group ceremonies; medicinal plants; herding; honey	Go deeper into forest to settle, herd, get timber; age group ceremonies; commercial timber business; medicinal plants
Development		ILIDP trains men and women in agriculture, organizes Field Days, women’s empowerment workshops	

In oral histories, older people described their lives as specialized pastoralists when they were young. Women used men’s age groups and intergroup violence as their temporal benchmarks.

We moved. We moved. My father was *Ilterito*. When he heard it had rained in another place we moved there for good grazing. I remember men burning a lot, so cows could get new grass without being disturbed by wild animals. Men drove the cows and we drove the sheep, goats and calves. We carried the little ones on our backs. We were just pastoralists. There was no cultivation. When *Ilkiseeyia* were warriors, *Sonjo* came and raided cows and killed an old man who was *Ilnyang’usi*. There was a *manyata* in Kipelian to fight back the *Sonjo*, so we were all moved to the *manyata* of those *Ilkiseeyia* warriors, so they could fight back the *Sonjo*. (FKI-NFE-83)^1^

Ninety-eight percent of household informants said men relied on women for house building in this period and up until just 10 years ago.

Men ordered us to go get building materials from the forest. If you didn’t do it, you were beaten. For us work was hard. We didn’t rest the whole day. We took the machete to the forest for building materials, tied the poles, and dragged them home like a prisoner. (FKI-NFE-59)

Women recalled their methods in rich detail.

When we were moving, we made houses of skin *(ilngoborri)* with only one bed. We didn’t stay in them a month. If we stayed longer, we made *manyata* houses out of dung. I went to the forest and cut *olmisigiyioi* and *olkinyei* strong enough to hold the skins. I planted sharp sticks, tied them with ropes, attached the skin on top, and we squeezed together to sleep. We are the last generation to live in skin houses. When not moving, I made a nice *manyata* house of dung. I measured where children will sit, where men will sit, and a place for the calves and young goats and sheep. I went to the forest for posts of *oloirien, olmisigiyioi, olgilai, olmaroroi, ormorijioi,* and *oledardar*. I used strings to tie the sticks on the roof. I carried as many as I could. …. In the morning, I put cow dung on the ground, so the posts were straight. I dug holes for center post of *olkinyei,* planted the four walls and tied the sticks around the posts. I plastered with cow dung. I went back to the forest for straight posts to make a nice bed and mat of *olsinoni* so it would not hurt me and the baby*.* (FKI-NFE-77)

All women said fuel wood gathering was their job then, as it still is today. Ninety-eight percent said this labor was lighter then, due to diet and resource availability.

When a woman went for firewood it would last two weeks. It was near because people were few. Cooking was little because we only cooked blood. There was no cultivation. When milk was plenty we drank it as food. When there was less, we drank blood and slaughtered cows for meat. Blood was harvested from the vein of a cow in a gourd, curdled, cooked in a pot, and served with a knife. We had it with tea as you would use cake or bread. Or water was put in a pot with milk and blood. Cow fat was added, and you drank it. It kept us alive*.* (FKI-NFE-59)

Older women had fond memories of periods known as *olpul* when, as uncircumcised girls *(intoyie),* they accompanied male peers (*moran*) to the forest for weeks of meat eating and drinking herbal soups infused with roots, leaves, and bark of specific forest plants for gendered purposes.

*…*The herbs boiled for men and girls to kill thirst were *olkiloriti, olkonyil, oledat, olkirenyi,* and *olakediai*. But that pot with *olangunguai,* girls were not allowed to drink. It was for moran to make them go crazy, to make them brave when they went stealing cows, hunting buffaloes, and killing lions. (FKI-NFE-83)

From 1976 to 1995, forest succession growth proceeded regionwide, notably in Empurputia where dense forest coverage increased from 23% to 46%. Over 60% of residents moved to the highland forest in Empurputia in this period. One elderly woman recalled how dark the forest was then: “We moved to this forest the year when *Ilbuluka* were circumcised. We feared the forest. The dark was continuous. You could not see a place which had light” (FKI-NFE-83).

This period coincided with some of the most severe droughts in Kenya’s modern history. All men said they permanently settled in Empurputia because of better grazing, and 23% also mentioned cultivation. Most women (60%) said they moved to marry. Taking advantage of the highland rain, women were the first to dig small gardens (*enkurma enkioni*) to feed their children. At the time, men did not think it was worthwhile for economic and cultural reasons.

When I was weeding my garden, my husband said, “You are doing nothing. Go look after my cows.” When he found me cooking vegetables he said, “Pour them out. What is this disgrace you are bringing by eating leaves?” I put the pot on top of the roof of the house so when he was away I could get it to cook and feed the children. (FKI-NFE-59)

The period also coincided with infrastructure development sponsored by a new non-governmental organization (ILIDP)^2^ that both men and women credited with training them in cultivation.

We were trained at Ilkerin to cultivate. Experts taught us the best seeds to plant and how to hold a *jembe* and train oxen. We saw the soil had importance, like cows*.* (MKI-NFE-48).

I went to the first field day at Ilkerin. Everybody went. People brought maize cobs and made them stand to be seen. If you had the biggest, you were given a machete or axe. We showed our beans, pumpkins, and tobacco. We were given a hoe. (FKI-NFE-59)

In the new cash economy, the small remunerations collected from vegetable sales comprised the main source of income for 18% of women, but 40% were still mainly dependent on husbands. The livestock economy dominated by men was still the main source of income for 92% of informants.

In the 1990s, the Narok County government was poised to change the forest’s legal status into a reserve for tourism purposes, wresting it from local control. To encourage elders to save the forest, women sang about the forest’s value. Elders recognized women’s agency in the forest conflict because the songs inspired men to persist in the decade-long legal battle.

I remember very well when women started singing those songs. They told leaders and the *oloiboni* not to allow the forest to be taken. The songs had a lot of power. I give them 8 out of 10*.* (MG-NFE-64)

The women’s songs focused heavily on medicinal plants.

We are from the Loita Forest where the grass is not sold. We boil *ormisigiyioi* for children and give them *oloirien* to embrace the culture*. Oleparmunyo* is the medicine for their colds. I can only compare it to a hospital. There is *olotoroniki* and *olkonyil* and pay attention to *orkitolosua*, the medicine for our moran when they go to slaughter oxen in the forest. If the dark forest inside is sold, cows will cry since their stomachs will be empty. Don’t give away the forest where morning comes slowly*.* (FKI-NFE-45)

This focus is noteworthy because only women gathered medicinal plants in the forest to treat children’s illnesses. They mentioned personal use of popular medicinal plants, such as *oleparmunyo* (a treatment for colds and malaria), more often than men (Fishers Test *p* = .004575, *N* = 60) and had more pessimistic predictions about its future availability (Wilcoxon Mann Whitney Test *p* = .00000866, N = 60). In transect walks, women pointed to a greater variety of plants they actively used (50 and 34 plants, respectively) and described more personal uses of plants than men did (95 and 65 uses, respectively).

From 1995 to 2014, land change patterns in Empurputia mirrored trends across Loita but at an exaggerated rate (Table 2, Fig. 2). Between 1979 and 2009, Loita population had tripled, and the fastest-growing area was the sub-location that included Empurputia (GoK 1981, 2010). Compared to 10–20 years ago, 97% of women and 87% of men said their lives were harder, but the impacts varied among women based on age, education, and activities. Gendered activities now had more immediate environmental impacts and mirrored the local drivers of tropical deforestation operating worldwide, i.e., wood extraction, infrastructure extension, and agricultural expansion (Geist and Lambin 2002).

Men took over women’s traditional house building role, felling timber, mostly *oltarakuai* and *olpiripiri,* with power saws to build permanent residences. Eighty-eight percent of household informants said only husbands and hired experts currently build new houses.

Today it is men who remove the houses from the forest. My son constructed this new house because I don’t know how. They construct houses that do not rain on you and the calves. You don’t wake up in rain and go out in the night to collect cow dung in the dark and climb on top and plaster. When you are married you are brought to the new house of timber already constructed for you. When you get a good house, which has been planted for you, has life not become good? (FKI-NFE-83)

Participatory mapping, participatory observation, and interviews corroborated that men travel deeper into the forest to find preferred timber trees compared to 10 years ago. Eighty-seven percent of household informants said traditional rules against the taking of live trees are broken more often. People predicted that by 2050 less than 10% of *oltarakuai* will remain, due mainly to men’s timber business.

In the past people were not using the forest for construction. There were no modern houses. Today the forest is a source of income. Men are eating it, using it for profit. Men are the forest eaters. Men sell timber and steal it at night. (MFC-SE-64)

People also acknowledged women’s impact from gathering the preferred fuelwood, *oloirien.* In participatory mapping, all women said they walked deeper into the forest to find it compared to 10 years ago. Participant observation and interviews showed that women harvest live trees (versus dead wood) using fire.

Women are very involved in forest destruction. They are burning live oloirien for firewood. The Forest Committee should make written rules and educate them about using dry branches, leaves or cow dung in gas stoves. (WG-HE-37)

*Oloirien* has many other uses and both men and women consider it the most preferable for ceremonial purposes and feeding cows in dry season. The women’s projection of its future availability in 2050 at less than 10% was more pessimistic than men’s (Wilcoxon Mann Whitney Test *p* = .0001185, *N* = 60).

Dependence on crop income has increased significantly (Table 4). The percentage of people cultivating (97%) was higher than other parts of Maasailand engaged in rain-fed cropping (Homewood *et al*. 2009) reflecting favorable environmental conditions and fewer opportunities for income diversification. Activity was concentrated along the forest edge, where people cleared to increase distance from trees where crop-raiding baboons live, and in the deeper forest where men felled *oltarakuai* for fencing*.*

The men described the shift to agro-pastoralism in relation to labor allocation.

We started cultivating because we needed a co-wife for the cow. A man takes a second wife because one alone cannot do all the milking, taking care of calves and sheep and children, weeding, and going for firewood and water. (MKI-SE-64)

As drought persisted, they diversified from pastoralism as their sole source of income.

The cows were not enough because of change in weather. We started moving outside and interacted with Kikuyu, Kalenjin, and guys from Somalia and Tanzania who worked in the maize fields. We wanted it for income. (MKI-SE-35)

Many informants (54%) remembered livelihood changing between 1995 and 2004, though most people over 40 (64%) remembered changes between 1985 and 1994 also. This represents a lag of at least a decade compared to Tanzanian Maasai in Ngorongoro District, where the peak of adopting cultivation was in the 1970s and progressed, as in Loita, from women’s small vegetable gardens to feed families to growing maize for income to save selling cows (McCabe *et al*. 2010). Men’s average plot size (4.2 acres) was significantly larger than women’s (1.5 acres) (Wilcoxon test *p* = .000002317, *N* = 56), and people understood the forest impact.

For us men of Empurputia we are busy extending our shambas so that they can be as big as 10 acres. We are not planning on how to protect the trees here. (MG-NFE-48)

Cultivation is destroying the forest since we are clearing forest for shambas by burning down trees. The trees have migrated to run away from Maasai. (MKI-SE-45)

Cultivation destroys the *oltarakuai* because it’s the one used for fencing shambas. Men cut it down with power saws today. They are finishing the forest. (FKI-NFE-77)

Landsat has limited capacity to differentiate patterns for cultivation (Campbell *et al*. 2005), especially on forest edges, and to distinguish small plots from surrounding grasslands (Baldyga *et al*. 2008). Small agricultural clearings in heterogeneous landscapes such as Loita Forest require high spatial resolution and algorithms that can account for differences in spectral reflectance that are not pronounced. Notwithstanding, positive trends in rain-fed subsistence cropping in Maasailand are well documented (Homewood *et al*. 2009); the patchy, diffuse spatial appearance of dense forest loss in this study has been associated with smallholder subsistence agriculture elsewhere (Geist and Lambin 2001), and the qualitative data indicated forest loss to cultivation.

Most men (63%) and women (69%) said they engaged in all cultivation duties from clearing to selling, but 83% said women were the main sellers of crops. This is probably because women depend more on crops to pay for food, clothing, and school fees. More men (90%) than women (54%) said their main crop was maize (the most commercially valuable crop) as women also grow beans, potatoes, spinach, and onions. Ninety percent of household crop income was from maize, with men averaging over three times women’s income ($296 and $90 per year, respectively). Male domination of crop income held for all crops, not only maize, as their larger plots generated four times as much income. In all, women retained about 24% of the total household crop income for their own expenses. No women in the household survey reported depending mainly on husbands for cash.

In the shift to cultivation women’s labor burdens had intensified.

In the past, our duties were those men told us to do. That is no longer. Today he builds the house, sires the children, looks for someone to herd, and then he goes. Women cultivate, do the herding, care for children, and spray the cows. There are many families where the husband doesn’t get money to pay for food or school fees. They have become wanderers and loiterers. Women just struggle on their own. Is not life for women harder? (FKI-NFE-83)

There was variability among households in how people perceived labor division. Some households reported spousal cooperation in generating income. In this household, the wife felt her status had improved.

We are strong in business and can get our own money and go to market and buy and sell. We have become important people in society because we cultivate and do business. (WG-NFE-35)

Today we do cultivation. We all go looking for our daily bread with our women. The man and cows are not the only ones to be depended on*.* (MG-NFE-48)

In addition to their traditional role in milking and caring for small stock, all wives were herding, spraying, injecting, deworming, tagging, taking cows to water or salt, slaughtering, butchering, and/or buying and selling livestock, including cows. Families were highly dependent on this labor because children were in school and men were often away.

Before a man might have 10 children and only one in school. So, the man could go look for the daily bread and the women stay at home. Today the women don’t stay at home because they do the herding of cows, goats, and sheep and look after the shamba. The children, who once gave them a rest, are all in school. (MFC-NFE-32)

Women’s livestock ownership did not equate with decision-making power because of the difference between (a) animals they owned and could decide to sell (*aitore*) and (b) those they considered theirs but needed permission to sell (*aitodol*). An example is a woman’s usufruct rights to cows allotted to her at marriage for her lifetime and to pass on to sons, but which she could not sell, slaughter, gift, or otherwise dispose of. When full ownership control over cattle assets was accounted for (*aitore*), the gender disparity index was 0.06 (428/7742—the ratio of the sum of women’s to men’s cattle assets that were weighted and adjusted by age of the cow). When women’s cow ownership was not accounted for (*aitodol*), the index was still low at only 0.18 (1372/7742). Other livestock index values were higher, e.g., sheep/goats (0.14), donkeys (0.63) and chickens (1.20). Chickens were the only animal women owned more of than men. Gender disparity for non-livestock livelihood assets was 0.28, an improvement based mostly on women’s ownership of cellphones, radios, flashlights, and garden tools.

Most women owning (*aitore*) livestock (60%) were leaders in women’s groups and school and church committees. In other groups called merry-go-rounds, a form of micro-financing where women pool money to buy and sell cows to pay for girls’ school fees, the women were educated, had their own sources of income, attended women’s empowerment trainings, and husbands helped with monthly dues. In Nvivo content analysis, both men and women mentioned education most often as a driver of cultural change.

In the past, women could not sell cows they owned. Because of education we are strong in business and can get our own money and go to market and buy and sell animals. In the past it was just by name you owned a cow. Your husband would take that cow in the night and you would not see a coin from it*.* (FKI-SE-40)

Key informants blamed overgrazing and settlement for the loss of grasslands (−21%) and wetland grasses (−60%). Sixty-two percent of herd owners said they have to go to the distant forest in dry season whereas just 10 years ago, 72% said there was always ample grass in the swamp valley. Field verification of geospatial data, informant recollections, and climate records (KMD 2014) confirmed the loss of wetland vegetation (as tall as 5 m) in this period. This decline was erroneously interpreted as forest loss in a global gaze dataset (Hansen *et al*. 2013).

The cows are increasing in Empurputia. They are grazing in the swamp and they step on it which makes the water go down. In the past, the cows were few and grazed on the edge of the swamp. You could not cross the swamp the water was so deep. The grass was so tall you could not see the elephants*.* (FKI-NFE-59)

Legal threats to communal land tenure of Loita Forest emerged in the 1990s, and a contentious debate about subdividing all of Loita continues to this day. Eighty-two percent of household informants were firmly against the idea of private subdivided land parcels. The main reasons were limited access to grazing (37%), concerns about selling to outsiders (16%), and forest destruction (13%). Women were more anxious than men about its effects on the family (Fishers test, *p* = .03253, *N* = 49), possibly based on news about negative effects of land tenure changes on women nearby in Maasai Mara (Bedelian and Ogutu 2017).

Subdivision is a bad idea. It will affect women because it will bring family conflict. Husbands will take the title deeds and sell the parcels to get money for drinking alcohol and leave children without land. (FKI-NFE-50)

## Discussion

These findings are inconsistent with oft-cited references to relatively undisturbed forest in Loita (Zaal and Adano 2012). Satellite data and interviews highlight dramatic land changes taking place in Loita highlands and support its characterization as an endangered ecosystem (KWS 2018) in a biodiversity hotspot (BirdLife International 2018; CEPF 2012). The rates of dense forest loss in the study area (−18%) and across Loita (−7%) are noteworthy in comparison with each other and other remotely sensed data indicating 3% forest loss (1990–2015) and 6% loss in tropical forests (1980–2010) worldwide (Keenan *et al*. 2015). They also contrast with reports of forest gain nationwide at an annual rate of 0.1% (from 6.01% forest cover in 2000 to 7.46% in 2015) (GoK 2016). These discrepancies may be partially accounted for by differing remote sensing methods and varying definitions of forest. Such issues have important resource policy implications as in the case of Kenya’s forest gain assessment as it works toward a target of 10% forest cover by 2030.

The negative association between dense forest and bush suggests ecological forest succession dynamics aligned with multiple variables including fire, climate, groundwater levels, wild herbivores, and anthropogenic influences. Forest gain from 1976 to 1995 likely ensued from the prevalent use of fire to establish grasslands in earlier decades (Talbot 1960) and the fire cycle of African pencil cedar (co-dominant canopy tree: Bussmann 2001; Maundu *et al*. 2001) allowing cedar to fill in and outcompete other species. Human pressure on the most preferred trees, *oltarakuai, olpiripiri,* and *oloirien,* which once represented dominant vegetation associations in Loita Forest (Bussmann 2002), is likely involved in the forest’s transformation between 1995 and 2014. Rainfall was certainly a variable in the doubling of wetland vegetation between 1976 and 1995 when impassable water levels and dangerous wildlife meant fewer people and minimal grazing. Such synergistic effects of people with non-anthropogenic variables have been observed in other wooded Maasai landscapes (Dublin 1995; Homewood and Rodgers 1991) making simplistic explanations about climax vegetation and ecological balance in this area very unlikely. The long-term norm in Loita is likely to be ever-changing oscillations in the forest/bush/grasslands/swamp mosaic with abiotic variables operating as potent underlying drivers.

Building on the work of Geist and Lambin (2002), gender appears as a cultural variable influencing the same proximate drivers of forest loss operating worldwide (wood extraction, agricultural expansion, infrastructure expansion). This study’s local-scale ground-truthing of wetland vegetation loss uncovered synergies between abiotic (climate) and anthropogenic (settlement, grazing, livelihood roles) variables that were invisible in the global gaze approach erroneously interpreting it as forest loss (Hansen *et al*. 2013). Lacking in situ local verification of the satellite signature for wetlands, coarse remote sensing methods can oversimplify the human/environment system and miss the progressive degradation of Kenya’s small wetlands (Mwita *et al*. 2013).

Given the small sample size, gender differences pertaining to house building, medicinal plants, fuelwood, cultivation, livelihood assets, and subdivision may reflect local realities only. Nonetheless, the triangulation of gender analysis with remote sensing methods offers a fresh perspective for other settings where gender simplifications about resource use persist. Variations in crop income among Maasai households are attributed to many factors (Homewood *et al*. 2009), but gender and intra-household dynamics are largely unaccounted for (Brockington 2001; Wangui 2008). With higher resolution satellite imagery to differentiate cultivated plots from low vegetation, linkages between land change and gendered roles, power relations, and income can become clearer. In Loita Forest, gender variation in who goes to the forest for what and when has direct relevance for gender-sensitive forest planning, with immediate attention needed for *oloirien*, an anti-malarial considered threatened with extinction (World Agroforestry Center 2011) and the severely locally threatened *oltarakuai* and *olpiripiri* (Maundu *et al*. 2001).

Remarkable transformations in land, livelihood, and culture coincided with perceived improvements in women’s social status. Pernicious gender inequities (e.g., 0.06 livestock asset disparity), however, make their improved status a notion that is real mainly in comparison to the past. Since the 1970s, women’s identity has expanded (a) beyond the house, since houses are no longer made by women with tree poles dragged from the forest edge but by men making money from deep forest timber they fell with power saws, (b) beyond milking and small stock, since women are household heads making livestock and herding decisions with husbands gone and children in school, (c) beyond water, milk, and fuelwood duties to maize fields and weekly markets where their free labor and crop income are critical to family well-being, and (d) beyond children around at home to schools where the women pay the fees with their own cash. The ambivalence about whether women’s lives are easier or harder today reflected individual circumstances based on intrahousehold power relations, age, and education. A few educated women with supportive husbands have taken a bold step away from the past in forming women’s groups where they own livestock, including cows. Elsewhere in Maasailand, members of women’s empowerment groups are experiencing similar benefits from the breadwinner effect (Goldman and Little 2015), suggesting this positive change in women’s status is not unique.

## Conclusion

These findings advance a new empirically based gender-inclusive narrative about land change and livelihood shift in Maasailand. They extend the meaning of the metaphor—*we needed a co-wife for the cow—*beyond adopting cultivation to remain pastoralists (McCabe *et al*. 2010) to the rising dependence on women’s labor and income for survival. They align with assertions that gender must constantly be reconstructed as men and women rework power relations when production systems change (Moore 1994). As land cover continues transforming in response to abiotic drivers and land tenure change, gender ideologies and oversimplifications will be counterproductive to pathways out of poverty and resource scarcity. Gender is a variable in land change dynamics that is worthy of attention.

## Footnotes

1 This notation characterizes informants by gender (M = male F = female), type of informant (KI = key informant, WG = women’s focus group, MG = male’s focus group, MFC = male forest committee member), level of schooling (NFE = no formal schooling, SE = secondary education, HE = higher education), and age.

2 Ilkerin Loita Integral Development Program was funded in the 1970s by the Catholic Diocese of Ngong and a Dutch Catholic agency (CORDAID).
